# Effectiveness of mindfulness-based interventions for people with dementia and mild cognitive impairment: A meta-analysis and implications for future research

**DOI:** 10.1371/journal.pone.0255128

**Published:** 2021-08-02

**Authors:** Maki Nagaoka, Zenta Hashimoto, Hiroyoshi Takeuchi, Mitsuhiro Sado

**Affiliations:** 1 Department of Neuropsychiatry, Keio University School of Medicine, Tokyo, Japan; 2 Department of Neuropsychiatry, Yokohama Municipal Citizen’s Hospital, Yokohama, Kanagawa, Japan; 3 Center for Stress Research, Keio University, Tokyo, Japan; Rutland Regional Medical Center, UNITED STATES

## Abstract

**Objective:**

To assess the effectiveness of mindfulness-based interventions on people with dementia and mild cognitive impairment.

**Methods:**

We searched several electronic databases, namely Cochrane Library, EMBASE, and MEDLINE with no limitations for language or document type (last search: 1 February 2020). Randomized controlled trials of mindfulness-based interventions for people with dementia and mild cognitive impairment compared to active-control interventions, waiting lists, or treatment as usual were included. Predefined outcomes were anxiety symptoms, depressive symptoms, cognitive function, quality of life, mindfulness, ADL and attrition. We used the random effects model (DerSimonian-Laird method) for meta-analysis, reporting effect sizes as Standardized Mean Difference. Heterogeneity was assessed with the I^2^ statistics.

**Results:**

Eight randomized controlled trials, involving 276 patients, met the eligibility criteria and were included in the meta-analysis. We found no significant effects for mindfulness-based interventions in either the short-term or the medium- to long-term on any outcomes, when compared with control conditions. The number of included studies and sample sizes were too small. Additionally, the quality of evidence was low for each randomized controlled trial included in the analysis. This is primarily due to lack of intent-to-treat analysis, high risk of bias, and imprecise study results. The limited statistical power and weak body of evidence prevented us from reaching firm conclusions.

**Conclusions:**

We found no significant effects of mindfulness-based interventions on any of the outcomes when compared with control conditions. The evidence concerning the efficacy of mindfulness-based interventions in this population is scarce in terms of both quality and quantity. More well-designed, rigorous, and large-scale randomized controlled trials are needed.

## Introduction

Dementia is a syndrome that can be caused by a number of progressive disorders that affect memory, thinking, behavior and the ability to perform everyday activities [1]. Furthermore, dementia affects psychological conditions; people with dementia often experience anxiety and depression. Although pharmacological treatments are commonly used for anxiety and depression in dementia, these approaches have limited evidence of benefit and can increase a risk of adverse events [2]. Especially, antipsychotics can cause significant adverse effects in people with dementia such as strokes, cerebrovascular adverse events and increased mortality, therefore these medications should be used only when nonpharmacological management has failed to provide benefit [3]. Given that pharmacological treatments exhibit limited efficacy and are associated with significant risk, there is an urgent need to develop effective non-pharmacological interventions. Fortunately, scientific evidence regarding the effectiveness of these interventions is growing. A review of psychological treatments for depression and anxiety in people living with dementia found promising evidence concerning the effectiveness of psychological therapies [4]. Several studies pertaining to the efficacy of psychosocial treatments found they positively affect cognition, quality of life as well as suppress neuropsychiatric symptoms and associated distress [5–7].

Mindfulness, among others, is one of the most prospective non-pharmacological interventions. It was first introduced in the 1980s by Kabat-Zinn to evaluate its utility in patients suffering from chronic pain; the results demonstrated substantial improvements in pain control and well-being [8]. Kabat-Zinn’s mindfulness program, mindfulness-based stress reduction (MBSR), has since been replicated in many other settings and various types of mindfulness-based interventions (MBIs) emerged in the following years, such as mindfulness-based cognitive therapy [9]. Since the introduction of mindfulness into clinical settings, many studies have documented its beneficial effects, such as improving psychological well-being [10, 11] and reducing anxiety, depression, and stress [12, 13] in both healthy and clinical populations. However, there is a paucity of meta-analytic evidence to examine those psychological interventions targeted at improving psychological distress and well-being in people living with dementia. The only MBI meta-analysis ever conducted [14] indicated that these interventions improve the depressive symptoms of dementia sufferers with statistical significance. The analysis, however, suffers considerable limitations relevant to the quality of evidence, including the type of studies included, and heterogeneity of the interventions. Specifically, nearly half of the included studies (four out of nine) were non-randomized controlled trials (Non-RCTs); furthermore, interventions distinct from MBIs were also included, such as the Kirtan Kriya meditation and Kundalini yoga interventions. This study aims to conduct a meta-analysis that follows a more rigorous protocol in order to explore the effectiveness of MBIs in improving the symptoms of anxiety and depression, cognitive function, and well-being of people with dementia and mild cognitive impairment (MCI).

## Materials and methods

### Inclusion criteria

#### Types of studies

Parallel-group, individual RCTs were included. We did not include cluster-randomized or crossover trials.

#### Types of participants

The participants included people with dementia and MCI. Dementia with Lewy bodies (DLB) and frontotemporal dementia (FTD) were excluded because the typical clinical symptoms observed in these diseases (e.g., visual hallucinations in DLB, personality change in FTD) differ considerably from those in Alzheimer’s disease or vascular dementia. Parkinson’s and Huntington’s disease were also excluded because these diseases produce a different clinical picture from common dementia; disorder of movement is more prominent than memory loss in these population.

#### Types of interventions

Studies were required to involve mindfulness meditation, either as adjunctive or as monotherapy. Studies testing other meditation interventions without reference to mindfulness, such as Kirtan Kriya, yoga, tai chi, and qigong were excluded. Mindfulness interventions that did not require formal meditation, such as acceptance and commitment therapy (ACT) were also excluded. We applied restrictions to the number, form, and length of MBI sessions; studies in which the MBI was delivered over more than four sessions, for one hour each, and in group settings were included.

Acceptable comparators included active-control interventions, waiting lists, or treatment as usual.

#### Types of outcome measures

The primary outcome was concerned with anxiety symptoms, while secondary outcomes pertained to depressive symptoms, cognitive function, quality of life, mindfulness, ADL, and attrition.

### Literature search and study selection

We identified trials for inclusion by searching electronic databases, including the Cochrane Library, EMBASE, and MEDLINE with no limitations for language or document type. We included all the studies that had been published before January 2020. The last search was conducted on February 1, 2020. The relevant search strategies are presented in S1 Appendix. Two authors (MN and ZH) independently screened the titles and abstracts of citations obtained during the literature searches. Citations judged as potentially eligible by one or both review authors were obtained as full text. Any disagreements were solved through deliberations or by consulting a third review author (MS).

### Assessment of risk of bias in included studies

The two authors (MN and ZH) assessed the risk of bias in accordance with the recommendations in Chapter 8 of the Cochrane Handbook for Systematic Review of Interventions [15]. The sources of bias include: selection bias, performance bias, detection bias, attrition bias, reporting bias, and intent-to-treat (ITT) analysis. We rated the risk of bias in each domain as either “high risk,” “unclear risk,” or “low risk” according to the Cochrane “Risk of bias” tool [16]. Any disagreements were solved through deliberations or by consulting a third author (MS). We requested any missing information related to the “Risk of bias” assessment from the original investigators.

### Quality of evidence: GRADE and “Summary of findings” tables

The quality of the body of evidence was assessed using the GRADE approach [17]; it was rated with the GRADE pro GDT software [18] for each major outcome. Two authors (MN and ZH) rated each domain separately for each comparison and resolved discrepancies by consensus. The four levels of rating (high, moderate, low, or very low) reflect the extent of our confidence that the point estimate of effect is correct, even when factoring in the risk of bias in the included studies, inconsistency between studies, indirectness in addressing our review question, imprecision of the effect estimate, ITT analysis, and publication bias.

### Data extraction

Two authors (MN and ZH) independently extracted data from eligible studies using a previously agreed-upon form. The collected information included the author names; publication dates; study design; eligibility criteria; the characteristics of the study population (including diagnosis, age, gender, education, race, and marital state); intervention characteristics (types of MBIs, dosage, and duration); types of comparator content; outcomes; and results. Regarding the results, we collected the outcome measures, time of assessment, and statistics (numbers of participants, means, and standard deviation). We entered data into Review Manager (RevMan) [Computer program] Version 5.3. Copenhagen: The Nordic Cochrane Centre, The Cochrane Collaboration, 2014 and checked it to ensure its accuracy. Any disagreements were solved either through deliberations or by consulting a third author (MS). When important information was not reported, we contacted the original investigators to obtain it.

### Data analysis

We used RevMan 5.3 for Windows to conduct data entries and calculate the effect sizes. Regarding dichotomous data, we used the risk ratio (RR)—with its 95% confidence interval (CI)—as the measure for treatment effect. For continuous data, we calculated the standardized mean difference (SMD) with 95% CIs. We assessed the statistical heterogeneity by visual inspection of forest plots, tests of significance level (P value), and the I^2^ statistics. We rated the level of heterogeneity across studies as either low (I^2^ = 25%), moderate (I^2^ = 50%), or high (I^2^ = 75%) [19]. We did not assess reporting biases since a minimum of 10 studies are required to meaningfully interpret funnel plots [20, 21].

### Data synthesis

We performed the meta-analyses only when we judged elements of the trials (including participants, interventions, comparisons, and outcomes) to be sufficiently similar, and when essential data was available. Due to a considerable amount of clinical heterogeneity across the included studies, we used the random effects model (DerSimonian-Laird method) to pool the results with inverse variance methods. With regard to studies reporting multiple measures for the same outcomes, we chose specific QOL measures for the meta-analysis, such as WHOQOL-OLD, rather than general ones. One study assessed cognitive functions using CAMDEX-R, which consists of MMSE and CAMCOG; we chose to employ the MMSE score since it is more often used in the included studies.

### Sensitivity analyses

Sensitivity analyses were performed to investigate the validity and robustness of the meta-analyses. The following sets of studies were separately analyzed for anxiety and depression: (a) dementia patients, (b) MCI patients, and (c) active control.

## Results

### Description of included studies

**Fig 1:**
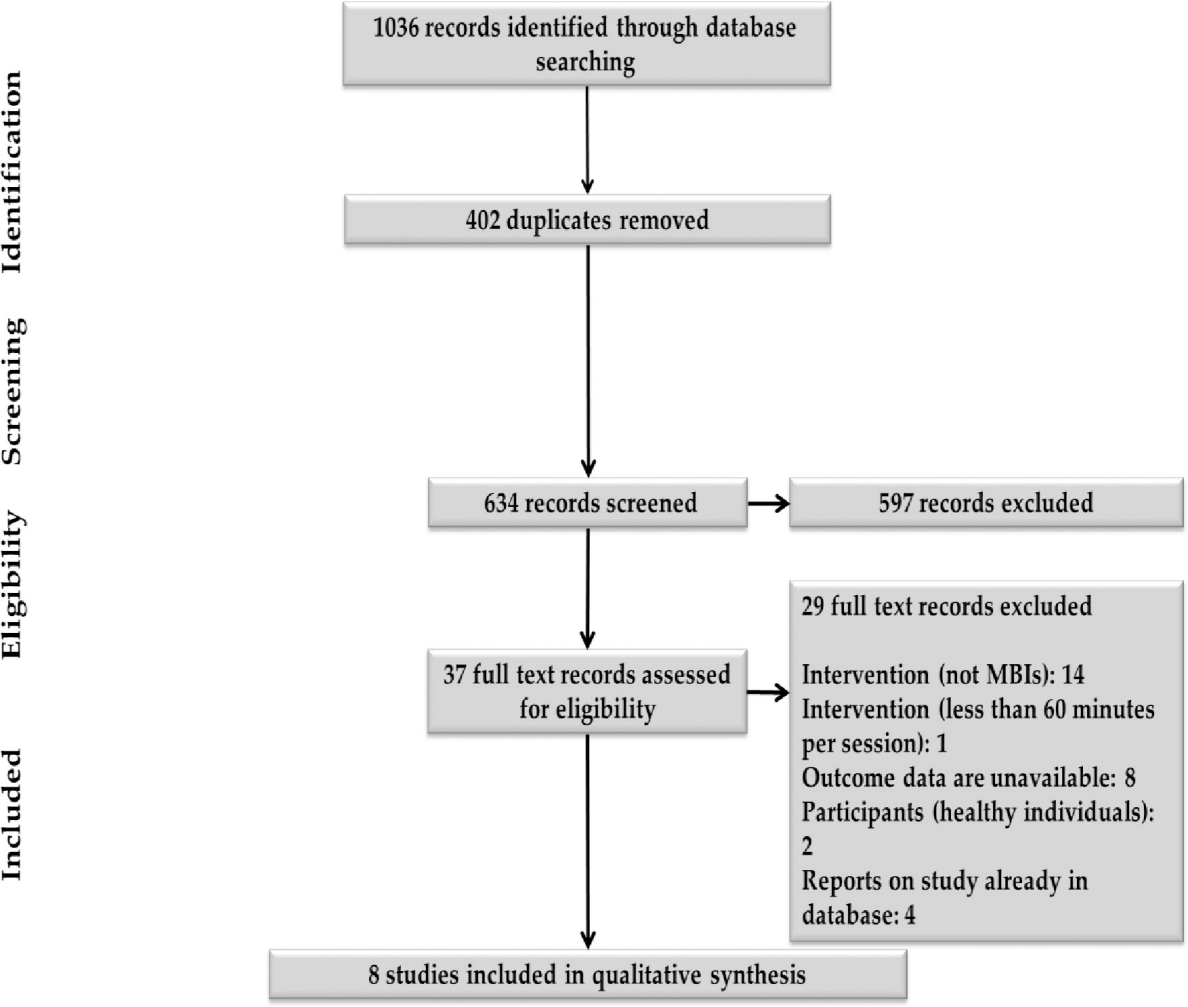
PRISMA flow diagram.

Our systematic literature searches identified 1036 records, of which 634 were left after removing duplicates (see Fig 1 for the PRISMA flow diagram). We excluded 597 records after a screening of the title/abstract. Accordingly, full-text articles were obtained for the 37 records identified as potentially eligible by two independent authors (MN and ZH): eight RCTs met inclusion criteria. Details concerning study characteristics are displayed in Tables 1 and 2

**Table 1:** Characteristics of included studies.

Study	Sample size	Country	Design	Population	Age (M (SD))	Intervention	Comparators
Chouinard, A. M., 2019 [22]	48	Canada	RCT	aMCI	MBI:72.7(7) control:70.7(5.6)	MBI for 8 weeks, 8 weekly sessions *2	Psychoeducation-based intervention
Churcher Clarke, A., 2017 [23]	31	UK	RCT	Dementia	80.61(9.4)	MBI for 5 weeks, 10 sessions, twice a week *1	Treatment as usual
Hanson, L. R., 2017 [24]	19	USA	RCT	Dementia	69.21(9.76)	Mindfulness program for 10 weeks, 10 weekly sessions *2	Psychoeducation
Larouche, E., 2016 [25]	22	Canada	RCT	MCI	71.6(7.6)	MBI for 8 weeks, 8 weekly sessions *2	Psychoeducation
Larouche, E., 2019 [26]	48	Canada	RCT	aMCI	MBI:71.4(7.7) control:70.5(5.6)	MBI for 8 weeks, 8 weekly sessions *2	Psychoeducation
Quintana Hernandez, D. J., 2016 [27]	85	Spain	RCT	Dementia	84.11(5.08)	MBSR + Kirtan Kriya for 2 years, 288 sessions *1 (this intervention is used in our analysis), cognitive stimulation, progressive muscle relaxation	Treatment as usual
Sheardova, K., 2019 [28]	28	Czech	RCT	MCI	Not reported	MBSR for 8 weeks *2	Cognitive games
Wells, R., 2013 [29]	14	USA	RCT	MCI	MBSR:73 (8) control:75 (7)	MBSR for 8 weeks, 8 session + 1 day retreat *2	Waitlist

*1: adjunctive therapy.

*2: unknown whether adjunctive or monotherapy.

Age (M (SD)), age mean (standard deviation); MBI, mindfulness-based intervention; MBSR, mindfulness-based stress reduction.

**Table 2:** Characteristics of included studies.

	Outcome measures						
Study	Anxiety	Depression	Quality of life	Cognitive function	Mindfulness	ADL	Attrition
Chouinard, A. M., 2019 [22]	GAI	/	/	/	/	/	〇
Churcher Clarke, A., 2017 [23]	RAID	CSDD	QOL-AD	MMSE	/	/	〇
Hanson, L. R., 2017 [24]	STAI	GDS	/	/	/	/	
Larouche, E., 2016 [25]	/	/	/	/	/	/	〇
Larouche, E., 2019 [26]	GAI	GDS	WHOQOL-OLD		FFMQ	/	〇
Quintana Hernandez, D. J., 2016 [27]	/	/	/	MMSE	/	RDRS-2	〇
Sheardova, K., 2019 [28]	/	/	/	/	/	/	〇
Wells, R., 2013 [29]	/	/	/	/	/	/	〇

GAI, Geriatric Anxiety Inventory; RAID, Rating Anxiety in Dementia Scale; STAI, State-Trait Anxiety Inventory; CSDD, Cornell Scale for Depression in Dementia GDS, Geriatric Depression Scale; QOL-AD, Quality of Life Alzheimer’s Disease scale; WHOQOL-OLD, World Health Organization Quality of Life Old scale; MMSE, Mini Mental State Examination; FFMQ, Five Facet Mindfulness Questionnaire; RDRS-2, Rapid Disability Rating Scale.

#### Study populations

The studies included a total of 276 participants; sample sizes ranged from 14 to 85. The mean age of participants ranged from 69.2 to 84.1.

The medical conditions that were reported in the studies included: dementia in three studies, MCI in three studies, and amnestic MCI in two studies.

#### Interventions

The total duration of the combined interventions ranged from 5 to 96 weeks; half of them (four studies) were eight weeks in length. In total, three studies were conducted on mindfulness-based stress reduction (MBSR) (one of them was on the combination of MBSR and Kirtan Kriya), and five on mindfulness-based interventions (MBI). Two studies used mindfulness as adjunctive therapy, and six did not state whether it was used as adjunctive or as monotherapy.

#### Comparators

Of the eight studies, two used treatment as usual as comparators, one used waiting lists, four used psychoeducation, and one used cognitive games as comparators.

#### Outcome measures

The analyses found that the most reported outcomes were anxiety symptoms. Continuous outcome data were reported by four studies that measured anxiety symptoms with certain scales, such as the Geriatric Anxiety Inventory (GAI) [30], the State-Trait Anxiety Inventory (STAI) [31], and the Rating Anxiety in Dementia Scale (RAID) [32]. Depressive symptoms were measured by three studies with the Geriatric Depression Scale (GDS) [33] and the Cornell Scale for Depression in Dementia (CSDD) [34]. A further two studies reported on the quality of life; one study used the Quality of Life Alzheimer’s Disease scale (QOL-AD) [35], while another used both the World Health Organization Quality of Life Old scale (WHOQOL-Old) [36]—specifically designed for assessing age-related quality of life—and the World Health Organization Quality of Life Brief scale (WHOQOL-Brief) [37]—which measures general quality of life. Cognitive function was reported in two studies; one study used the Mini Mental State Examination (MMSE) [38] and the other used the Revised Cambridge Examination for Mental Disorders of the Elderly (CAMDEX-R), which consists of MMSE and the Cambridge Cognitive Examination (CAMCOG) [39]. Only one study reported mindfulness and ADL outcomes using the Five Facet Mindfulness Questionnaire (FFMQ) [40] and the Rapid Disability Rating Scale (RDRS-2) [41], respectively.

#### Excluded studies

We excluded 29 studies after full-text screening. The main reason for exclusion was that the interventions were not MBI; detailed reasons are outlined (see Fig 1).

### Study quality and risk of bias

The quality of the body of evidence was assessed with respect to each outcome. We determined that most studies exhibited low quality. Details of the quality ratings are displayed in the “Summary of findings” tables (S1 and S2 Tables).

The risk of bias was judged for all the included studies. Regarding selection bias, five studies were categorized as exhibiting “unclear risk” due to insufficient information about the sequence generation process, thus not permitting a judgement of “low risk” or “high risk.” All the included studies were categorized as exhibiting ‘unclear risk’ with regard to allocation concealment for the same reason as random sequence generation. For performance bias, all studies were rated as “high risk” since, given the nature of psychosocial interventions, it is impossible to blind personnel or participants (or both). Furthermore, for detection bias, we determined whether outcome assessors were blinded to allocation; four studies were rated as “unclear risk.” For attrition bias, we appraised the amount, nature, and handling of incomplete outcome data. Two studies were rated as “high risk” because they reported different proportions of missing data across groups, and applied inadequate statistical methods for handling missing data, such as analyzing complete cases. Regarding reporting bias, we searched for protocols of the included trials, and subsequently established whether all the outcomes listed in the protocols were reported in the trials. We categorized all the studies as exhibiting “unclear risk” for the unavailability of protocols. For other biases, we rated four studies as being “high risk” due to the absence of ITT. An overview of our judgements about each “Risk of bias” item for individual trials and across all trials is outlined in S1 Fig.

### Effects of interventions

The results of each outcome, excluding attrition, are presented in short-term time frames (six weeks to ten weeks after the beginning of the intervention) as well as medium- to long-term time frames (11 weeks to six months after the beginning of the intervention) in the following sections.

#### Attrition

**Fig 2:**
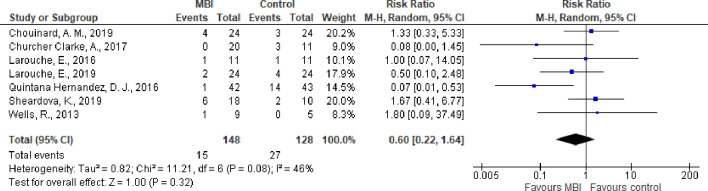
Forest plot of comparison: Attrition, MBI versus control.

Only seven trials reported data on attrition from treatments that could be meta-analyzed. Due to the very low quality of evidence, it remains unclear whether there was a lower risk of attrition in the MBI group than in the controls: RR 0.60 (95% CI 0.22 to 1.64; P = 0.32; 7 trials; 276 participants) (Fig 2) (S1 Table).

#### Anxiety symptoms

**Fig 3:**
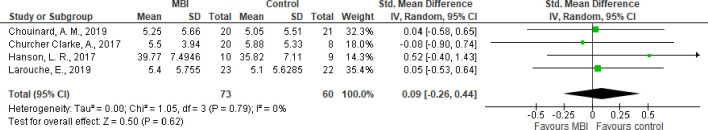
Forest plot of comparison: Anxiety symptoms, MBI versus control after 6 to 10 weeks from the beginning of the intervention.

**Fig 4:**
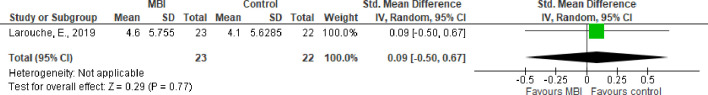
Forest plot of comparison: Anxiety symptoms, MBI versus control after 11 weeks to 6 months from the beginning of the intervention.

Regarding short-term effects, four trials exhibited continuous outcome data after measuring anxiety symptoms that could be meta-analyzed. Pooling these effects in a random-effects meta-analysis demonstrated that there were no clear differences in the effects on anxiety symptoms in the MBI group when compared to the control groups: standardized mean difference (SMD) 0.09 (95% confidence interval (CI) -0.26 to 0.44; P = 0.79; four trials; 133 participants) (Fig 3). We determined that the quality of evidence was very low (S1 Table). Furthermore, regarding the medium- to long-term effects, only one trial reported continuous outcome data on anxiety symptoms. There were no clear differences in the effects on anxiety symptoms in the MBI group, when compared to the control groups: SMD 0.09 (95% CI -0.50 to 0.67; P = 0.77; one trial; 45 participants) (Fig 4). We rated the quality of evidence as low (S2 Table).

#### Depressive symptoms

**Fig 5:**
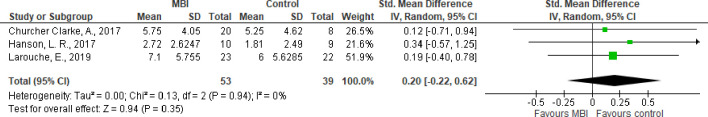
Forest plot of comparison: Depressive symptoms, MBI versus control after 6 to 10 weeks from the beginning of the intervention.

**Fig 6:**
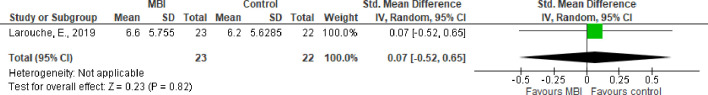
Forest plot of comparison: Depressive symptoms, MBI versus control after 11 weeks to 6 months from the beginning of the intervention.

Three trials reported the short-term continuous outcome data on depressive symptoms. There were no clear differences in the effects on depressive symptoms in the MBI group, when compared to the control groups: SMD 0.20 (95% CI -0.22 to 0.62; P = 0.35; three trials; 92 participants) (Fig 5). Again, we deemed the quality of evidence to be very low (S1 Table). For medium- to long-term effect, one trial reported continuous outcome data. However, due to the low quality of evidence, we were uncertain whether MBI had any effects on depressive symptoms, when compared to the control groups: SMD 0.07 (95% CI -0.52 to 0.65; P = 0.82; one trial; 45 participants) (Fig 6) (S2 Table).

#### Cognitive function

**Fig 7:**
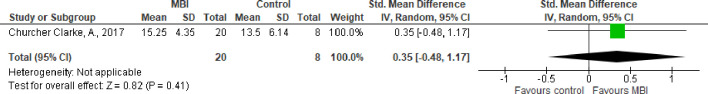
Forest plot of comparison: Cognitive function, MBI versus control after 6 to 10 weeks from the beginning of the intervention.

**Fig 8:**
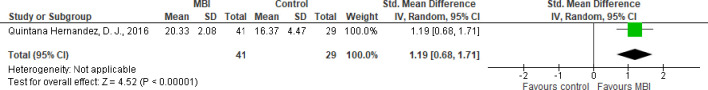
Forest plot of comparison: Cognitive function, MBI versus control after 11 weeks to 6 months from the beginning of the intervention.

One trial reported short-term outcomes while another reported medium-to-long-term outcomes on cognitive functions. Due to the very low quality of evidence, we were uncertain whether MBI had any different effect on cognitive functions—either in the short-term or the medium- to long-term time intervals, when compared to the control groups: short-term, SMD 0.35 (95% CI -0.48 to 1.17; P = 0.41; one trial; 28 participants) (Fig 7) (S1 Table), medium- to long-term, SMD 1.19 (95% CI 0.68 to 1.71; P<0.001; one trial; 70 participants) (Fig 8) (S2 Table).

#### Quality of life

**Fig 9:**
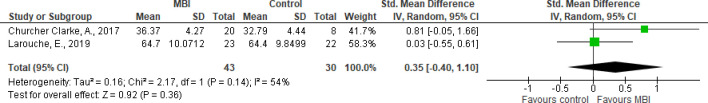
Forest plot of comparison: Quality of life, MBI versus control after 6 to 10 weeks from the beginning of the intervention.

**Fig 10:**
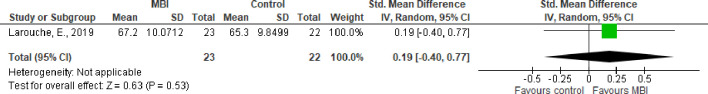
Forest plot of comparison: Quality of life, MBI versus control after 11 weeks to 6 months from the beginning of the intervention.

Two trials reported that short-term continuous outcomes, related to the quality of life, could be meta-analyzed. However, since the quality of evidence was very low, we were uncertain whether MBI had any different effects on quality of life, when compared to the controls: SMD 0.35 (95% CI -0.40 to 1.10; P = 0.36; two trials; 73 participants (Fig 9) (S1 Table). Furthermore, although one trial did report medium- to long-term quality of life outcomes, we were uncertain whether MBI had any different effects on the quality of life, when compared to the controls due to the low quality of evidence: SMD 0.19 (95% CI -0.40 to 0.77; P = 0.53; one trial; 45 participants) (Fig 10) (S2 Table).

#### Mindfulness

**Fig 11:**
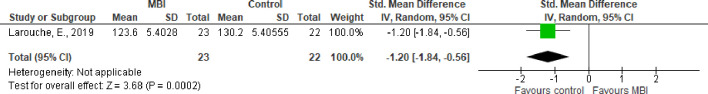
Forest plot of comparison: Mindfulness, MBI versus control after 6 to 10 weeks from the beginning of the intervention.

**Fig 12:**
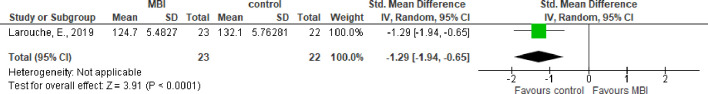
Forest plot of comparison: Mindfulness, MBI versus control after 11 weeks to 6 months from the beginning of the intervention.

While one trial reported the short-term continuous outcome data, we were still uncertain whether MBI had any different effects on mindfulness, when compared to the control groups since the quality of evidence was low: SMD -1.20 (95% CI -1.84 to -0.56; P<0.001; one trial; 45 participants) (Fig 11) (S1 Table). Likewise, a trial reported medium- to long-term data, but for the same reason as with the short-term, we were uncertain whether MBI had any different effects on mindfulness, when compared to the control groups: SMD -1.29 (95% CI -1.94 to -0.65; P<0.001; one trial; 45 participants) (Fig 12) (S2 Table).

#### ADL

**Fig 13:**
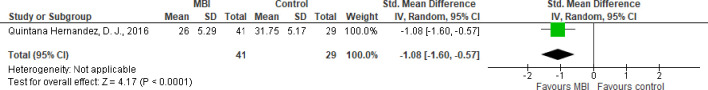
Forest plot of comparison: ADL, MBI versus control after 11 weeks to 6 months from the beginning of the intervention.

None of the included trials reported any short-term effects on this outcome. As to the medium-to-long-term effect, one trial did report continuous outcome data on ADL. Nevertheless, we were uncertain whether MBI had any different effect on ADL, when compared to the control groups sine the quality of evidence was very low: SMD -1.08 (95% CI -1.60 to -0.57; P<0.001; one trial; 70 participants (Fig 13) (S2 Table).

### Sensitivity analyses

None of the sensitivity analyses resulted in any significant differences between the groups. We restricted the first sensitivity analysis to the studies concerning dementia patients; the results indicated that there were no significant differences between the MBI and the control groups in anxiety or depressive symptoms. The second sensitivity analysis was restricted to the studies concerning MCI patients; the results also demonstrated that there were no differences between the groups regarding anxiety and depressive symptoms. The third sensitivity analysis was restricted to the studies concerning active controls; it resulted in no significant differences between groups in either anxiety or depressive symptoms. Details of the sensitivity analyses are displayed in S2 Fig.

## Discussion

This is the first meta-analysis that examines the effects of MBIs which uses data solely from RCTs on the attrition, mental health, cognitive function, mindfulness, and quality of life of older people with dementia.

### Overall results and comparison with other studies

Overall, the results revealed no significant effects for MBIs in either short-term or medium- to long-term time intervals on any outcomes, when compared with control conditions.

We explore three of the outcomes in this discussion: anxiety, depression, and mindfulness. We particularly focused on these outcomes because anxiety and depression are the most common psychological symptoms among people living with dementia, while mindfulness represents the essential outcomes of MBIs and relates to the improvement of other clinical symptoms.

It is well known that dementia is associated with an increase in anxiety. This is reflected in that the prevalence of anxiety among elderly healthy people is 2.9%, but this rises among people with dementia to 14.0% [42]. Therefore, efficacious anxiety treatments for those with dementia is imperative. Unfortunately, the MBIs exhibited no superiority over control conditions. Conversely, a recent review indicated that MBIs do improve anxiety in older adults without cognitive impairment [43]. This inconsistency could imply that MBIs for people with dementia may require some modifications to account for the related characteristics (e.g., cognitive impairments, attention deficit, and concentration). Shortening the time for each session, simplified instructions, and reminding patients of the time for practicing meditation at home, for example, might be of help for future research regarding MBIs for people with dementia. Another possible explanation for the discrepancy might relate to a mere lack of statistical power. As shown in Fig 3, the samples included in the analysis totaled at 133. This number is quite small when compared to the analysis, with a sample of 418 adults, which showed MBIs’ effectiveness on anxiety [44]. In order to ensure an adequate sample size to assess MBIs’ effectiveness, further RCTs that target people with dementia are needed.

Similar to the case of anxiety, the prevalence of depression among people with dementia is higher than among community samples (25% vs 12.8%) [42]. Consequently, we found no significant effects of MBIs on depressive symptoms. However, this finding contradicts the results of the meta-analysis by Strauss [44] regarding young to middle-aged adults with depressive symptoms, which revealed that MBIs are effective for people with a current depressive disorder. Similar to the anxiety analysis, we posit two explanations for this gap: modifications for MBIs are needed to account for the characteristics of people with dementia, and the lack of statistical power (n = 92 in total). However, comparing our results to those of Wang’s study [14], a meta-analysis of MBIs for people with dementia, might cause confusion because it confirms the efficacy of MBIs in improving the depressive symptoms of people with dementia. We assert that this discrepancy is due to the following reasons. The first pertains to the difference in the type of studies included. In Wang’s study [14], non-RCTs as well as RCTs were included in the analysis, while our meta-analysis only included data from RCTs. The second reason is concerned with the different MBI definitions. We focused exclusively on MBIs that place emphasis on mindfulness meditation as a core feature of the intervention. Conversely, in Wang’s study [14], interventions in which mindfulness meditation is not placed as a core component, such as Kirtan Kriya meditation or Kundalini yoga, were also included. These differences may have affected the discrepancy in the results. Regardless, since we focused solely on RCTs targeting MBIs that position mindfulness meditation as a core feature, the results of this study are more precise and of greater value when evaluating MBIs’ effectiveness on depressive symptoms.

With respect to the mindfulness outcome, it is surprising that unfavorable results were observed for MBIs when compared to the control groups. However, the results at the endpoint might have been affected by a baseline difference since the mean for mindfulness (FFMQ) in the controls was higher than in the MBIs already at baseline (MBIs mean123.1 (SD5.34), control mean = 126.1 (SD5.98); favorable for the control already at baseline). Furthermore, mindfulness is supposed to be the first improvable outcome for MBIs. Garland et al., [45] proposed The Mindfulness-to-Meaning Theory which indicates that mindfulness practice improves mindfulness skills, which leads to increasing metacognitive capacity for experiences and results in an increase in positive affect. This theory was verified by a previous RCT which indicated that improving mindfulness skills mediated the effect of an intervention on clinical outcomes. [46]. Therefore, the unfavorable results of the MBIs in this analysis might indicate the difficulty of improving mindfulness in people with dementia. If this is the case, consideration should be given to adjusting the mindfulness program to fit the characteristics of people with dementia. Finally, it is critical to note that only one out of the eight studies included in the current meta-analysis measured mindfulness as a clinical outcome. We were, therefore, unable to perform an effective meta-analysis. Since the mindfulness is the core component of MBIs, as previously mentioned, its measurement should be a focus in all related research.

### Challenges in the field of MBIs for people with dementia

The results of this meta-analysis revealed several challenges in the field of MBIs for people with dementia. First, studies exploring the effectiveness of MBIs in people with dementia are still in their infancy, there were few studies to include. Furthermore, the sample sizes are very small; five of the studies randomized fewer than 40 participants. Therefore, the statistical power is limited. Second, the quality of RCTs really matters. Unfortunately, the quality of evidence of the RCTs included in the analysis were rated from low to very low; this was primarily due to reasons like a lack of ITT, high risk of bias, and imprecision of study results. Consequently, the weak body of evidence prevented us from reaching firm conclusions. Third, most of the included studies did not measure mindfulness, despite it being an outcome with a great likelihood of improvement. As a result, we were unable to determine how MBIs influence mindfulness ability.

### Implications for future research

In light of the challenges mentioned above, future research should address the following issues. First, they should consider the quality of trials. More well-designed, rigorous, and large-scale RCTs are needed to provide precise information regarding the effectiveness of MBIs for people with dementia. The included studies suffered from many limitations. For example, four of the eight studies did not conduct ITT analyses, which are necessary to draw accurate and unbiased conclusions regarding the effectiveness of an intervention. Therefore, employing ITT analysis is highly recommended for future research. Second, studies should enroll samples large enough to detect statistical differences in the outcomes, more specifically a minimum of 40 participants. Third, in order to assess long-term effects, studies should conduct follow-ups for 6 to 12 months. Fourth, since the rationales for MBIs is to improve mindfulness skills, mindfulness should be measured as a clinical outcome. Fifth, since the effectiveness of MBIs largely depends on program content and the competency of mindfulness instructors, both factors should be reported in future studies.

### Limitations

First, the limited statistical power due to the small sample size of the studies included prevented from detecting statistical differences in the outcomes. Second, although the control groups included various conditions, we could not conduct subgroup analyses to sort them because of the insufficiency of the included trials. Third, even though the extent to which patients benefit from MBIs would be related to the severity of dementia, we could not conduct subgroup analyses because the severity is unknown in most of the studies included. Fourth, the MBIs included in the analysis exhibit heterogeneity to some extent in terms of their content and duration. Especially for small meta-analysis like ours, levels of undetected heterogeneity are likely to be high, therefore the results may be affected by this factor. Fifth, because we were unable to generate funnel plots on account of the limited available data, we cannot exclude the possibility of publication bias. In general, publication bias is smaller in meta-analyses of more recent studies [47], older studies included in the present study could be more biased.

## Conclusions

This is the first meta-analysis based solely on RCTs that examined the effectiveness of MBIs on the mental health, cognitive function, mindfulness, and quality of life of older people with dementia. We found no significant effects of MBIs on any of the outcomes when compared with control conditions. Bearing in mind the small number of studies included, their small sample size and low quality of evidence as well as the possibility of publication bias, the results of our meta-analysis need to be interpreted with caution. Nevertheless, the results of this study have shed light on some challenges: evidence concerning the efficacy of MBIs in this population is scarce in terms of both quality and quantity. More well-designed, rigorous, and large-scale RCTs are needed.

## Supporting information

S1 Checklist (DOC)

S1 Fig Risk of bias assessment. (DOCX)

S2 Fig Sensitivity analyses. (DOCX)

S1 Table Summary of findings (6w-10w). (DOCX)

S2 Table Summary of findings (11w-6m). (DOCX)

S1 Dataset (XLSX)

S1 Appendix Search strategies. (DOC)
