# Supplementary material for: Effectiveness of mindfulness-based interventions for people with dementia and mild cognitive impairment: A meta-analysis and implications for future research
Source: PLoS One. 2021 Aug 2;16(8):e0255128. doi: 10.1371/journal.pone.0255128 (PMC8328308; doi:10.1371/journal.pone.0255128)
Supplement: S1 Fig — (DOCX) [file pone.0255128.s002.docx]

**Risk of bias graph: review authors’ judgements about risk of bias item presented as percentages across all included studies.**


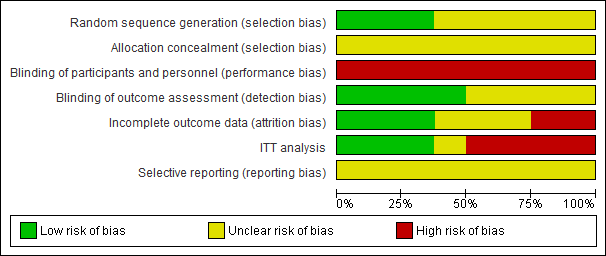


**Risk of bias summary: review authors’ judgements about each risk of bias item for each included study.**

**
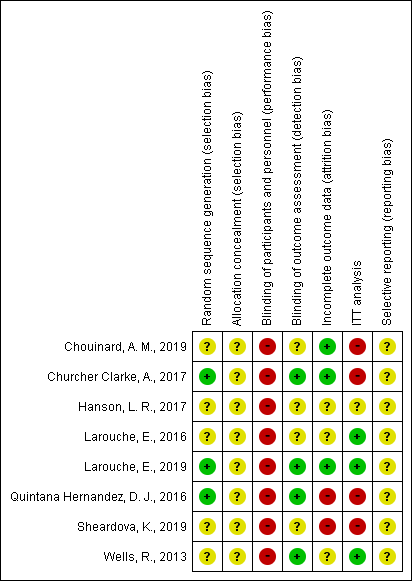
**
