# Supplementary material for: Effectiveness of mindfulness-based interventions for people with dementia and mild cognitive impairment: A meta-analysis and implications for future research
Source: PLoS One. 2021 Aug 2;16(8):e0255128. doi: 10.1371/journal.pone.0255128 (PMC8328308; doi:10.1371/journal.pone.0255128)
Supplement: S2 Fig — (DOCX) [file pone.0255128.s003.docx]

**S2 Fig. Sensitivity Analyses**

**Anxiety symptoms 6-10w. (a) Dementia patients**


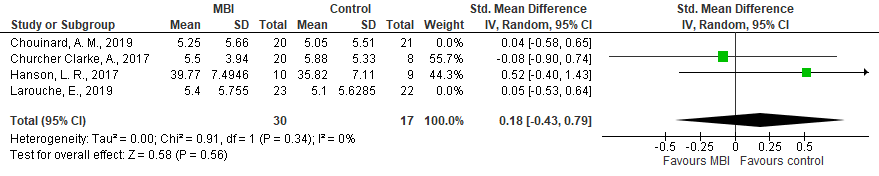


**Anxiety symptoms 6-10w. (b) MCI patients**


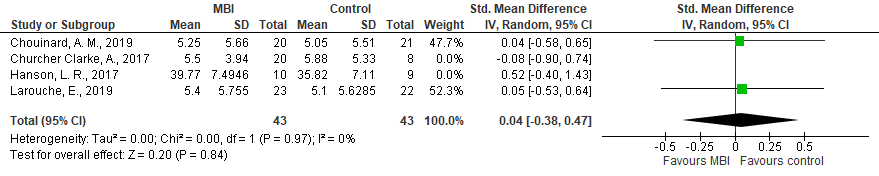


**Anxiety symptoms 6-10w. (c) Active controls (psychoeducation)**

**
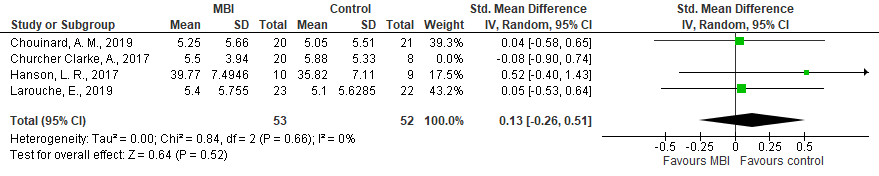
**

**Depressive symptoms, 6-10w**. **(a) Dementia patients**


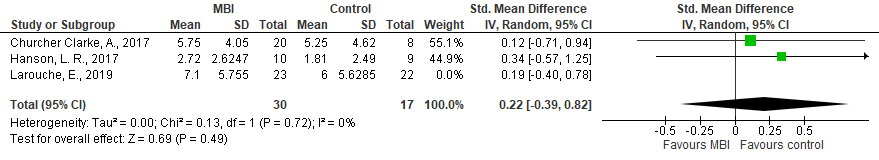


**Depressive symptoms, 6-10w**. **(b) MCI patients**


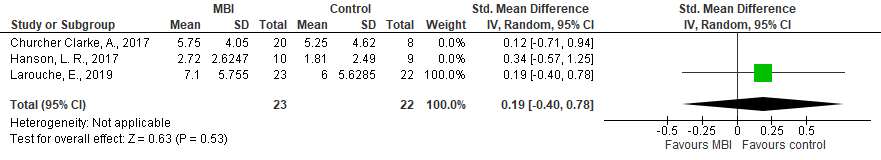


**Depressive symptoms 6-10w. (c) Active controls (psychoeducation)**

**
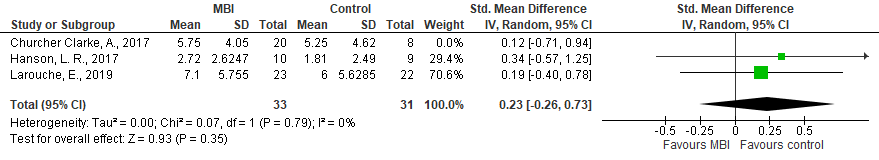
**
