# Supplementary material for: Effectiveness of mindfulness-based interventions for people with dementia and mild cognitive impairment: A meta-analysis and implications for future research
Source: PLoS One. 2021 Aug 2;16(8):e0255128. doi: 10.1371/journal.pone.0255128 (PMC8328308; doi:10.1371/journal.pone.0255128)
Supplement: S2 Table — (DOCX) [file pone.0255128.s005.docx]

**SUMMARY OF FINDINGS**

**Question**: MBI compared to control for dementia

| **Certainty assessment** | | | | | | | **№ of patients** | | **Effect** | | **Certainty** | **Importance** |
| --- | --- | --- | --- | --- | --- | --- | --- | --- | --- | --- | --- | --- |
| **№ of studies** | **Study design** | **Risk of bias** | **Inconsistency** | **Indirectness** | **Imprecision** | **Other considerations** | **MBI** | **control** | **Relative (95% CI)** | **Absolute (95% CI)** |  |  |
| **Cognitive Function (11W~6M)** | | | | | | | | | | | | |
| 1 | randomised trials | very serious ^a,b,e^ | not serious | not serious | serious ^c^ | none | 41 | 29 | - | SMD **1.19 higher** (0.68 higher to 1.71 higher) | ⨁◯◯◯ VERY LOW ^a,b,c,e^ |  |
| **Depression (11W~6M)** | | | | | | | | | | | | |
| 1 | randomised trials | serious ^b^ | not serious | not serious | serious ^c^ | none | 23 | 22 | - | SMD **0.07 higher** (0.52 lower to 0.65 higher) | ⨁⨁◯◯ LOW ^b,c^ |  |
| **Anxiety (11W~6M)** | | | | | | | | | | | | |
| 1 | randomised trials | serious ^b^ | not serious | not serious | serious ^c,d^ | none | 23 | 22 | - | SMD **0.09 higher** (0.5 lower to 0.67 higher) | ⨁⨁◯◯ LOW ^b,c^ |  |
| **QOL (11w~6m)** | | | | | | | | | | | | |
| 1 | randomised trials | serious ^b^ | not serious | not serious | serious ^c,d^ | none | 23 | 22 | - | SMD **0.19 higher** (0.4 lower to 0.77 higher) | ⨁⨁◯◯ LOW ^b,c^ |  |
| **Mindfulness(11w~6m)** | | | | | | | | | | | | |
| 1 | randomised trials | serious ^b^ | not serious | not serious | serious ^c^ | none | 23 | 22 | - | SMD **1.29 lower** (1.94 lower to 0.65 lower) | ⨁⨁◯◯ LOW ^b,c^ |  |
| **ADL (11w~6m)** | | | | | | | | | | | | |
| 1 | randomised trials | very serious ^a,b,e^ | not serious | not serious | serious ^c^ | none | 41 | 29 | - | SMD **1.08 lower** (1.6 lower to 0.57 lower) | ⨁◯◯◯ VERY LOW ^a,b,e^ |  |

**CI:** Confidence interval; **SMD:** Standardised mean difference; **RR:** Risk ratio

#### Explanations

a. We downgraded the quality of evidence by one level due to serious concern about high risk of bias in ITT analysis.

b. We downgraded the quality of evidence by one level due to serious concern about high risk of bias in blinding of participants and personnel.

c. We downgraded the quality of evidence by one level due to insufficient sample size.

d. We downgraded the quality of evidence by one level due to imprecision of confidence intervals which includes potential for harm or benefit

e. We downgraded the quality of evidence by one level due to serious concern about high risk of bias in incomplete outcome data.
