# Supplementary material for: Effectiveness of mindfulness-based interventions for people with dementia and mild cognitive impairment: A meta-analysis and implications for future research
Source: PLoS One. 2021 Aug 2;16(8):e0255128. doi: 10.1371/journal.pone.0255128 (PMC8328308; doi:10.1371/journal.pone.0255128)
Supplement: S1 Appendix — (DOC) [file pone.0255128.s007.doc]

# **S1 Appendix: Search strategies**

**I: Review Title**

The effectiveness of the third wave Cognitive Behavioral Therapies for elderly patients with dementia

**II: Search Date**

February 1, 2020

**III: Limitations for Search**

Date/Time: No

Language: No

Document Type: No

Publication Status: Published

**IV: Resources and Number of Results**

**Table 1** Search resources details and number of results

| **Resource** | **Time Coverage** | **Search Interface** | **# of Hits** |
| --- | --- | --- | --- |
| Cochrane | Until Search Date | Cochrane Library | 209 |
| EMBASE | 1974 – 2019 October 04 | Ovid SP | 527 |
| MEDLINE | 1946 to January 16, 2020 | Ovid SP | 300 |
|  | | | |
| Subtotal | 1036 | | |
| Duplicates | 402 | | |
| **Total (for Screening)** | **634** | | |

S1 **Appendix: Search Strategies**

***A. Cochrane Library***

#1 MeSH descriptor: [Dementia] explode all trees 5807

#2 MeSH descriptor: [Alzheimer Disease] explode all trees 3351

#3 MeSH descriptor: [Dementia, Vascular] explode all trees 351

#4 (dement* or alzheimer*):ti,ab,kw (Word variations have been searched) 19433

#5 ("cognitive impairment" or "cognitive decline" or DLB or MCI or aaci or cind or arcd or acmi or "n-mci" or "a-mci" or "m-mci" or nmci or amci or mmci):ti,ab,kw (Word variations have been searched) 10472

#6 #1 or #2 or #3 or #4 or #5 25677

#7 MeSH descriptor: [Mindfulness] explode all trees 721

#8 ((third NEAR/1 wave NEAR/2 (CBT or cognitive))):ti,ab,kw (Word variations have been searched) 27

#9 (compassion NEAR/3 therapy):ti,ab,kw (Word variations have been searched) 57

#10 ("integrative therapy"):ti,ab,kw (Word variations have been searched) 123

#11 (mindfulness* OR MBCT OR MBSR OR "activity scheduling" or meditation or "mind-body"):ti,ab,kw (Word variations have been searched) 5908

#12 #7 or #8 or #9 or #10 or #11 6083

#13 #6 and #12 209

***B. EMBASE***

1 exp dementia/ (350917)

2 exp alzheimer disease/ (195278)

3 exp multiinfarct dementia/ (12056)

4 (dement* or alzheimer*).tw,kw. (304192)

5 (cognitive impair* or cognitive decline or DLB or MCI or aaci or cind or arcd or acmi or "n-mci" or "a-mci" or "m-mci" or nmci or amci or mmci).tw,kw. (141447)

6 or/1-5 (482547)

7 exp mindfulness/ (7605)

8 (third adj wave adj2 (CBT or cognitive)).tw,kw. (97)

9 (compassion adj3 therap*).tw,kw. (150)

10 integrative therap*.tw,kw. (636)

11 (mindfulness* or MBCT or MBSR or activity schedul* or meditation or "mind-body").tw,kw. (17547)

12 or/7-11 (19588)

13 6 and 12 (527)

***C. MEDLINE***

1 exp dementia/ (161207)

2 exp alzheimer disease/ (91213)

3 exp dementia, vascular/ (6517)

4 (dement* or alzheimer*).tw,kw,kf. (212996)

5 (cognitive impair* or cognitive decline or DLB or MCI or aaci or cind or arcd or acmi or "n-mci" or "a-mci" or "m-mci" or nmci or amci or mmci).tw,kw,kf. (86832)

6 or/1-5 (298011)

7 exp mindfulness/ (2890)

8 (third adj wave adj2 (CBT or cognitive)).tw,kw,kf. (66)

9 (compassion adj3 therap*).tw,kw,kf. (122)

10 integrative therap*.tw,kw,kf. (380)

11 (mindfulness* or MBCT or MBSR or activity schedul* or meditation or "mind-body").tw,kw,kf. (12798)

12 or/7-11 (13557)

13 6 and 12 (300)
